# Stimulation of Midbrain Dopaminergic Structures Modifies Firing Rates of Rat Lateral Habenula Neurons

**DOI:** 10.1371/journal.pone.0034323

**Published:** 2012-04-02

**Authors:** Xuefeng Shen, Xiaoguo Ruan, Hua Zhao

**Affiliations:** Department of Physiology, Norman Bethune College of Medicine, Jilin University, Changchun, Jilin, China; University of Chicago, United States of America

## Abstract

Ventral tegmental area (VTA) and substantia nigra pars compacta (SNpc) are midbrain structures known to be involved in mediating reward in rodents. Lateral habenula (LHb) is considered as a negative reward source and it is reported that stimulation of the LHb rapidly induces inhibition of firing in midbrain dopamine neurons. Interestingly, the phasic fall in LHb neuronal activity may follow the excitation of dopamine neurons in response to reward-predicting stimuli. The VTA and SNpc give rise to dopaminergic projections that innervate the LHb, which is also known to be involved in processing painful stimuli. But it's unclear what physiological effects these inputs have on habenular function. In this study we distinguished the LHb pain-activated neurons of the Wistar rats and assessed their electrophysiological responsiveness to the stimulation of the VTA and SNpc with either single-pulse stimulation (300 µA, 0.5 Hz) or tetanic stimulation (80 µA, 25 Hz). Single-pulse stimulation that was delivered to either midbrain structure triggered transient inhibition of firing of ∼90% of the LHb pain-activated neurons. However, tetanic stimulation of the VTA tended to evoke an elevation in neuronal firing rate. We conclude that LHb pain-activated neurons can receive diverse reward-related signals originating from midbrain dopaminergic structures, and thus participate in the regulation of the brain reward system via both positive and negative feedback mechanisms.

## Introduction

A great deal of research has been focused on the role of midbrain dopaminergic system in the regulation of reward. For example, studies have reported that phasic changes in the activity of dopamine neurons in the ventral tegmental area (VTA) and substantia nigra pars compacta (SNpc) specifically predict reward probability [1], [2], [3]. The LHb, in particular its medial portion (LHbM), projects to and receives dopaminergic projections from the VTA and SNpc via the fasciculus retroflexus [4], [5], [6], [7]. Its role in the reward system has therefore also been a focus of much recent research.

In addition to its role in the reward system, the LHb has been implicated in a number of related functions, including depression and pain sensitivity. Both depression and pain are associated with negative reward functions and are also linked to the dopaminergic system [8], [9], [10]. Thus, decreasing LHb neuronal activity has been reported to improve behavior in a rat model of depression [11] and also to reduce symptoms of depression in some patients [12], [13], [14]. There are a number of pain-activated (PA) neurons in the LHb [15], [16], [17] and the stimulation of LHb causes a decrease in pain thresholds [18].

One hypothesis related to the role of LHb in reward is that transient elevation in the firing rate of LHb neurons encodes negative reward value [3], [19], because it rapidly induces the inhibition of firing in midbrain dopamine neurons, whose activity is linked to positive reward value [3], [20], [21]. Similarly, the LHb stimulus played a profound inhibitory role of dopamine release in the nucleus accumbens [22], a key component of the reward system [23], [24]. These findings support the idea that the LHb is a source of negative reward signals, acting by inhibiting midbrain dopamine neurons.

An interesting phenomenon is that transient activation of midbrain dopamine neurons evoked by reward-predicting stimuli may occur before the transient inhibition of LHb neural activity [3], suggesting that positive reward signal from the dopamine neurons may be conveyed to the LHb neurons. However the role of the reciprocal midbrain dopaminergic projection to the LHb is less clear. Local application of dopamine [25] increased the activity of LHb neurons. Similarly, systemic administration of dopaminergic agonists generated increases in firing of LHb neurons [26], [27], [28], which is inconsistent with mutually inhibitory relations between the LHb and SNpc/VTA. The latter two manipulations, however, involved tonic dopaminergic input to the LHb, suggest that transient activation of SNpc/VTA is associated with transient suppression of LHb neuronal activity. Thus, transient and sustained input from midbrain dopaminergic systems to LHb may have opposite functional effects on LHb neurons. But this proposition has not been tested with electrophysiological study. Therefore, in this study we investigated the impact of two patterns of electrical stimulation (single-pulse and tetanic stimulation) of the VTA and SNpc on firing rates of LHb PA neurons, thus to identify an intact neural circuit with functionally between the LHb and midbrain dopaminergic structures.

## Results

A total of 80 PA neurons were recorded in the LHb, including 46 cells in the medial and 34 cells in the lateral divisions. The average firing rates of these PA neurons were increased from 6.7±0.8 Hz to 11.7±1.1 Hz in response to tail pinch stimulation (*P*<0.0001). Fifteen animals underwent lesions of the fasciculus retroflexus before the recording session; these accounted for 22/31 recordings in the LHbM and 9/31 recordings in the lateral portion of the LHb (LHbL). Stimulating electrodes were implanted in a total of 24 animals in the VTA (44 cells recorded in the LHb) and 17 animals in the SNpc (36 cells recorded in the LHb) (Table 1).

**Table 1:** Distribution of recorded LHb PA neurons.

	Intact		Lesioned		All
	LHbM	LHbL	LHbM	LHbL	
**VTA**	12	17	10	5	44
**SNpc**	12	8	12	4	36
**All**	24	25	22	9	80

### Effects of stimulating the VTA on the firing of PA neurons in the LHb

**Figure 1:**
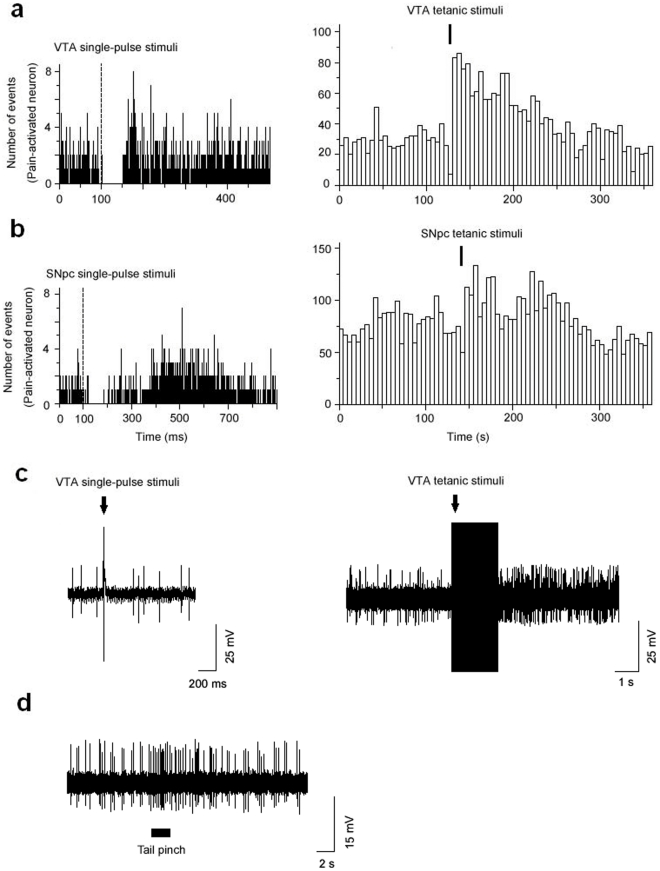
Effects of single-pulse stimulations of the VTA and the SNpc on the LHb PA neurons. The recordings of panels a, b and c are from different neurons. (a, b, c left panels) The LHb PA neurons generally exhibited a similar transient cessation in firing in response to the single-pulse VTA stimulation and the single-pulse SNpc stimulation. The left panel c showed an original firing recording for the LHb PA neuron's response to single-pulse stimulations of the VTA. Both the peristimulus time histograms were comprised of 100 consecutive sweeps. Each single stimulus pulse was delivered 100 ms after the onset of each sweep (bin: 1 ms; a, b left panels). (a, b, c right panels) PA neuron firing was increased by the tetanic VTA stimulus and the tetanic SNpc stimulus. The right panel c showed an original firing recording for the LHb PA neuron's response to the tetanic VTA stimulus. Both histograms displayed using a bin of 5 s (a, b right panels). (d) an original firing recording for the LHb PA neuron's response to tail pinch.

**Figure 2:**
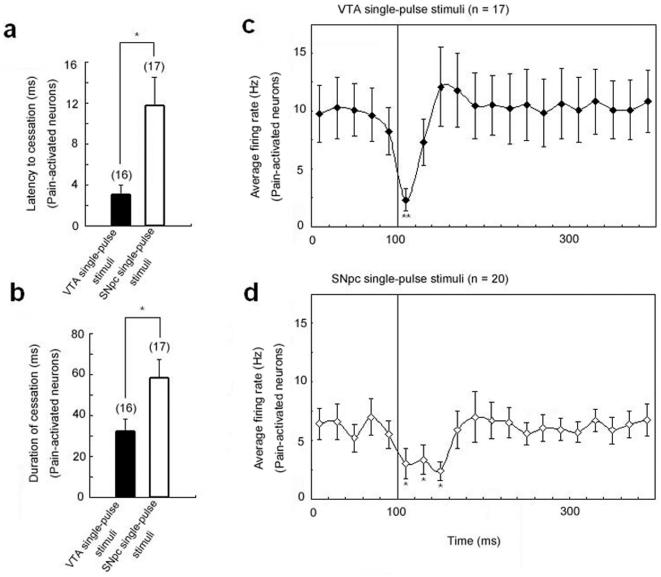
Comparisons between responses of LHb PA neurons to the single-pulse stimuli of respective dopaminergic structures. (a) The latency to onset of the cessation induced by the SNpc stimulus is notably longer than that induced by the VTA stimulus. (b) The duration of cessation in firing of PA neurons in response to the SNpc stimuli seems much more marked than the one in response to the VTA stimuli. Black asterisks indicate a significant difference (*P*<0.05). (c, d) Average activity of LHb PA neurons during single-pulse stimulation of the VTA (filled rhombus, n = 17) and the SNpc (open rhombus, n = 20), respectively. Single (*P*<0.005) and double black asterisks (*P*<0.0001) indicate a marked firing suppression compared with baseline (0 ms to 100 ms). Error bars indicate SEM.

As in previous studies [29], [30], [31], single-pulse stimulation of the VTA (300 µA, 0.5 Hz) was used to mimic a transient dopamine signal at the level of the LHb. Data were obtained from 17 identified PA neurons in the LHb (baseline firing rate, 6.9±1.8 Hz), of which 16 showed complete suppression of firing with short latency (mean, 3.0±1.0 ms) after VTA stimulation (Fig. 1a.), and 1 failed to respond to stimulation. Cessation of firing activity persisted for an average of 31.8±6.5 ms (range, 9–109 ms; Fig. 2c).

The effect of tetanic VTA stimulation (80 µA, 25 Hz) was investigated in 25 PA neurons: 10 in the medial division and 15 in the lateral division of the LHb. Tetanic stimulation of the VTA evoked marked increases in firing in 13/25 PA neurons (Fig. 1a), 8/10 in the LHbM and 5/15 in the LHbL. Ten PA neurons did not respond to tetanic VTA stimulation and 2 showed reductions in firing rates. Mean firing rates of the 13 activated PA cells increased from 11.0±3.2 Hz to 15.8±4.0 Hz in response to tetanic VTA stimulation (*P*<0.0005), with an average latency of 6.7±3.6 s and a duration of 43.3±18.2 s, and mean firing rates of all 25 PA neurons also increased from 10.6±2.2 Hz to 12.9±2.6 Hz (*P*<0.01).

The proportion of PA cells showing activation in response to tetanic VTA stimulation was significantly higher in the LHbM than in the LHbL (*P*<0.05; Table 2). This result is consistent with evidence that the VTA innervates primarily the LHbM division [7].

**Table 2:** Region-specific characteristic of activated LHb PA neurons to tetanic VTA stimuli.

	LHbM	LHbL	All
**Activation**	8	5	13
**Suppression**	1	1	2
**No response**	1	9	10
**All**	10	15	25

Due to limited number of the PA neurons which showed suppression in firing, these numbers are added to those present no response to the VTA stimulus during performing the *χ* ^2^ test.

Thirteen of these 25 PA neurons were tested with both single-pulse and tetanic VTA stimulations. 6/13 cells were activated, 1/13 was suppressed, and the others did not respond to the tetanic VTA stimulation, but all of the 13 cells showed phasic fall in firing in response to the single-pulse VTA stimulus.

### Effects of stimulating the SNpc on the firing of PA neurons in the LHb

Single-pulse stimulation of the SNpc at the same current parameters as used in the VTA (300 µA, 0.5 Hz) also induced transient suppression of firing in 17/20 PA neurons (baseline firing rate, 6.1±1.3 Hz); 3 were activated. The latency to onset of firing-rate suppression averaged 11.7±2.8 ms (Fig. 1b), significantly longer than for VTA stimulation (3.0±1.0 ms; *P*<0.05; Fig. 2a). The mean duration of firing suppression was 58.1±9.4 ms (range, 16–119 ms; Fig. 2d), which was also significantly longer than the response to VTA stimulation (31.8±6.5 ms; *P*<0.05; Fig. 2b).

Tetanic SNpc stimulation effects were assessed in 11 LHb PA neurons. Of these, 5 showed increased firing (from 5.2±3.1 to 9.2±3.4 Hz; *P*<0.05) (Fig. 1b), 2 showed decreased firing, and 4 did not respond. The average firing rate of all 11 cells was not changed significantly (5.6±1.8 Hz versus 7.5±2.2 Hz; *P*>0.05).

All 11 PA neurons on which were applied tetanic SNpc stimuli also got single-pulse stimuli of the SNpc. No matter how the PA cells responded to the tetanic SNpc stimulus, all of these cells showed a transient firing cessation in response to the single-pulse SNpc stimulus.

### Effects of fasciculus retroflexus lesions on LHb responses to VTA and SNpc stimulation

**Figure 3:**
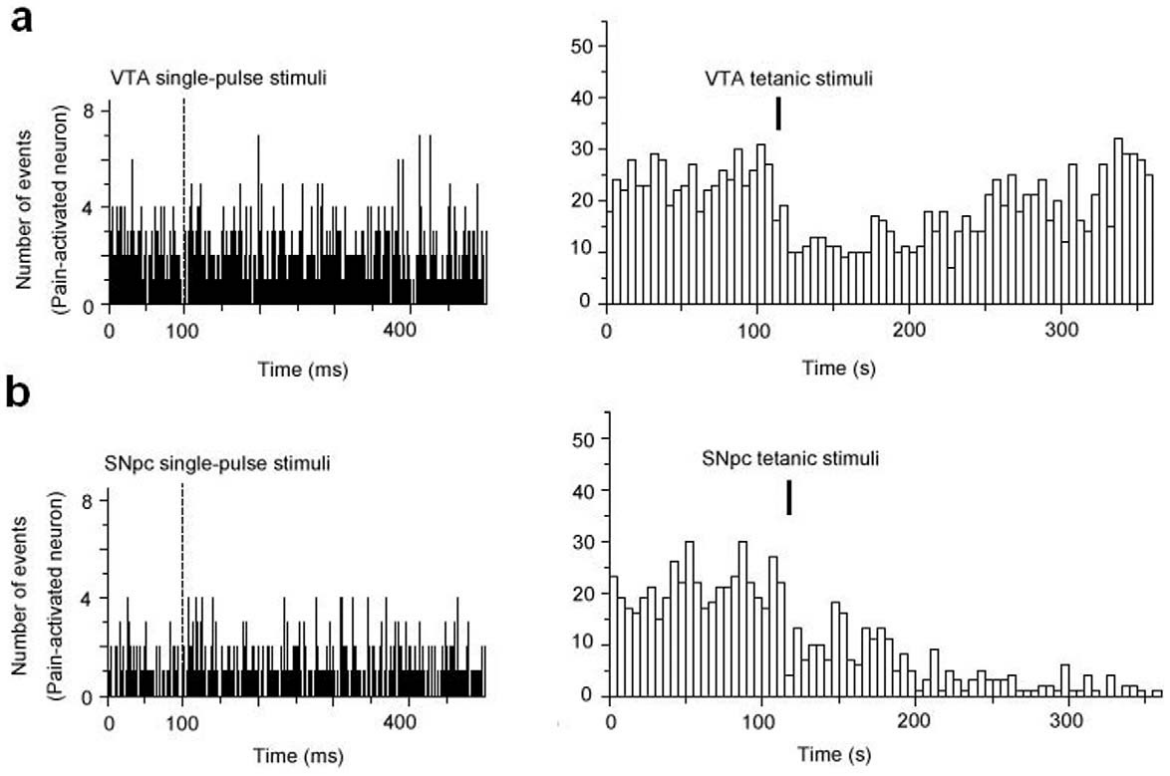
Electrolytic lesions of the fasciculus retroflexus. (a) Intact bilateral fasciculus retroflexus are shown in this panel. (b) Electrolytic lesions caused complete lesions (n = 9; average area proportion >90%). (c) Partial lesions were found in some animals (n = 6; average area proportion >60%). fr, fasciculus retroflexus; ml, medial leminscicus; D3V, dorsal third ventricle; 3V, third ventricle.

We next assessed the effect of lesions of the fasciculus retroflexus, the principal pathway for dopaminergic projections reaching the LHb [6]. Electrolytic lesions were applied on 26 rats in total, 15 of which were identified as successfully lesioned models, including 9 with complete lesions (Fig. 3b) and 6 with partial lesions (Fig. 3c). Data obtained from unsuccessfully lesioned models were not included.

**Figure 4:**
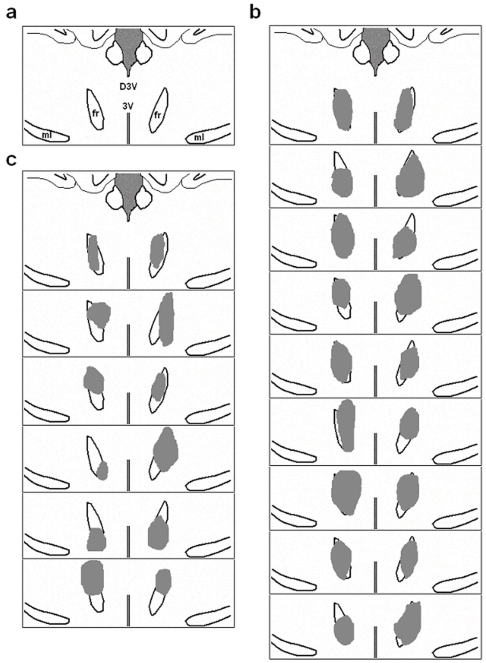
Effects of the fasciculus retroflexus lesions on LHb PA neuron firing. (a, b left panels) The bilateral lesions attenuated the rapid inhibitory effect of the single-pulse VTA and SNpc stimulation on the LHb PA neurons. Peristimulus time histograms were compiled from 100 consecutive sweeps and each single stimulus pulse was delivered at 100 ms (bin: 1 ms). (a, b right panels) Firing rates of these pain-activated neurons recorded in the VTA and SNpc stimuli groups were suppressed after the lesions (bin: 5 s).

After lesions of the fasciculus retroflexus, 11/15 of PA neurons failed to respond to single-pulse stimulation in the VTA (Fig. 4a), while 1/17 of LHb PA neurons failed to respond to the VTA stimulation in intact animals (*P*<0.005). Four of 15 of PA neurons still showed a firing cessation in response to the single-pulse VTA stimulus (15.0±5.5 ms), which was slightly shorter than that recorded in intact animals (31.8±6.5 ms; *P*>0.05). 8 rats were used in this part. These 4 cells were obtained from 2 rats with complete lesions and 1 rat with partial lesions.

Eight of thirteen of PA neurons did not respond to single-pulse stimulation of the SNpc (Fig. 4b), while 1/29 of LHb PA neurons failed to respond to the SNpc stimulus in intact animals (*P*<0.01). Mean firing cessation of 4/13 of PA neurons in response to the single-pulse SNpc stimulation (17.5±2.7 ms) was notably shorter than that observed in intact animals (58.1±9.4; *P*<0.05). The rest 1 cell was activated. 7 rats were used in this part. These 5 cells that still responded to the stimulus were obtained from 1 rat with complete lesions and 4 with partial lesions.

Five of fifteen PA neurons firing in the LHb showed suppression (8.0±3.1 Hz versus 5.1±1.7 Hz; *P*>0.05) in response to the tetanic VTA stimulation after the lesions (Fig. 4a). Seven cells failed to respond to the stimulus, and 3 other cells showed firing activation. The mean spontaneous firing rates of these 15 cells before the VTA stimulus was not different significantly in the comparison with that after the VTA stimulus (9.5±2.1 Hz versus 9.5±2.4 Hz; *P*>0.05) with lesions of the fasciculus retroflexus. Of 8 neurons which responded to tetanic stimulus of the VTA, 5 neurons failed to respond to single-pulse stimulus of the VTA and the others showed phasic fall in firing.

Five out of nine PA neurons exhibited suppression in firing (5.2±2.3 Hz versus 2.2±1.7 Hz; *P*<0.05) (Fig. 4b), one cell showed activation in firing, and the remainder failed to respond to the tetanic SNpc stimuli after the lesions. The difference was not found in the comparison of the mean spontaneous firing rates of these 9 cells before and after the SNpc stimulus (5.6±1.6 Hz versus 4.1±1.5 Hz; *P*>0.05). Four of six cells that responded to the tetanic SNpc stimuli also got single-pulse stimuli, but 3 of these 4 cells still showed the changes of phasic fall in firing.

## Discussion

Although the LHb has been demonstrated to receive dopaminergic fibers from midbrain VTA and SNpc [4], [5], [6], [7], key components of the reward system, it still remains unclear how LHb neurons respond to the signals from these dopaminergic structures. Recent research based on the reward-biased visual saccade task reported that the inhibition of LHb neurons followed the excitation of dopamine neurons elicited by reward-predicting stimuli [3], suggesting that the transient inhibitory signal in the LHb may be from the excitatory action of midbrain dopamine neurons.

Single-pulse stimulation is used to simulate the transient reward signal in this study. The majority of PA neurons in the LHb exhibited a phasic fall in firing in response to the VTA stimuli. Moreover, SNpc stimuli caused a more marked suppression (58 ms) in PA neuron firing than the VTA stimuli (32 ms), with a longer latency (12 ms for SNpc stimuli versus 3 ms for VTA stimuli), at the same stimulation parameters. Although neuroanatomical research revealed that the dopaminergic projection of SNpc to LHb is sparser than that of VTA to LHb, the dopaminergic ratio of the SNpc projection may be higher than the VTA one [6]. It appears likely phasic dopamine transmission plays an inhibitory role on the LHb PA neurons. Single-pulse stimulation of the SNpc may lead to an intensified phasic dopamine release in the LHb that contributes to a stronger inhibition of these PA neurons. Alternatively, the SNpc stimulation may influence the interneurons in LHb which link to PA neurons directly, and thus plays a delayed but stronger inhibitory role on these cells. However the electrical stimulation of the VTA and SNpc may also activate non-dopaminergic neurons (e.g. GABAergic neurons) and fibers of passage in addition to dopaminergic neurons and the effect might be involved in the induction of phasic fall in firing of PA neurons.

The effect of single-pulse stimulation of the VTA and SNpc on the firing of LHb PA neurons was significantly blocked by electrolytic lesions of the bilateral fasciculus retroflexus in the present research. It is congruous with the previous study that the density of dopaminergic nerve terminals in the LHb is weakened after lesions of the fasciculus retroflexus [5]. These findings suggest that the transmission of positive reward signal from the dopaminergic system to the LHb is dependent on the intact fasciculus retroflexus. Matsumoto and Hikosaka reported that there is a delayed phase between the excitation of dopamine neurons and the following inhibition of LHb neurons in reward trials [3], suggesting that the suppression of LHb firing may be induced by the excited dopamine neurons as mentioned above. Congruously, our data show that the single-pulse midbrain stimulus plays an inhibitory role on the PA neurons in LHb. The excitation of LHb correlated to the negative reward signal encoding [3], [19], [32], which suggests that the inhibition of LHb is crucial to achieving reward activity. Moreover, suppressed activity of LHb could elevate dopamine release in the downstream targets of dopaminergic system, such as nucleus accumbens [22]. Ji and Shepard reported that electrical stimulation of LHb had an inhibitory effect on the firing activity of dopamine neurons in VTA and SNpc [21]. Thus, the phasic inhibitory effect on the PA neurons may rapidly evoke the disinhibition of dopamine neurons and prolong the excitatory phase of dopamine neurons. It suggests that the LHb is crucial to maintain the efficiency of reward signal encoding through this positive feedback process.

Habenular nucleus, especially LHb, has been shown as a pain regulation center in brain, where large numbers of PA neurons are distributed [16], [17]. Recent research shows that most of the LHb PA neurons exhibit inhibition and excitation in response to rewarding and aversive stimuli, respectively [19], suggesting that the PA neurons (excited by aversive stimuli) might be the principle type of neurons in the LHb involved in encoding the negative reward rule. In addition, relief of painful sensation could be also considered as rewarding effect [10]. Thus, the PA neurons in LHb were inhibited by the midbrain stimuli in our experiments, which is consistent with the reward coding theories [9]. It suggests that the inhibition of LHb PA neurons might be one component of positive reward signal encoding.

The inhibition of LHb neuron firing induced by single-pulse stimuli of the VTA and SNpc is consistent with the behavioral research that the excitation of dopaminergic neurons is induced by reward-predicting stimuli and is followed by the inhibition of LHb neurons [3]. However, recent studies show that systemic application of dopaminergic agonists increases the activity of most of LHb neurons *in vivo* [26], [27], [28]. Micro-injection of dopamine into the LHb markedly reduced 5-HT release in the midbrain via activating the LHb [25]. There is a similar report that the firing of LHb PA neurons is enhanced by micro-electrophoresis of cocaine into the LHb [33]. These findings show that the application of dopamine in the LHb induces not only an inhibitory response of its neurons, but also excitatory response, potentially depending on the level of dopamine neurons in the LHb or the level of the VTA and SNpc activated.

In this study the tetanic stimulation of the VTA evoked the excitation of approximately half of the PA neurons in the LHb. The stimulating electrodes were implanted in the anterior VTA in our experiments based on previous work showing that dopaminergic fibers in the LHb are primarily from the anterior VTA [7]. We note that of the proportion of PA neurons affected, the excitation is higher in the medial LHb, the major area receiving dopaminergic fibers in LHb [7]. These findings indicate that the VTA-induced excitation of PA neurons might be attributed to the action of VTA dopamine release in the LHb.

The PA neurons in LHb showed an excitatory response to tetanic stimulus, an opposite effect to phasic inhibitory response induced by single-pulse stimulus in our recording. The two different physiological effects induced by tetanic and single-pulse stimulus may be associated with D_1_ and D_2_ receptor activated, respectively, in the LHb [34], [35] because activated D_1_ and D_2_ receptors, respectively, are able to induce excitatory and inhibitory of adenylate cyclase via Gs (stimulatory) protein and Gi (inhibitory) protein coupled respectively [36], [37]. However it will be involved in our further study.

Forty-seven percent and 33% of PA neurons did not show any response to tetanic VTA and SNpc stimuli, respectively, after lesions of the bilateral fasciculus retroflexus. However 33% and 56% of PA neurons in the LHb have an inhibition to tetanic VTA and SNpc stimulation respectively after the lesions. The bilateral fasciculus retroflexus lesions reversed the excitatory effect to the inhibitory effect on the PA neurons induced by the tetanic VTA and SNpc stimuli, suggesting that tetanic stimuli of the dopaminergic structures may trigger an alternative pathway except for the fasciculus retroflexus. Presumably, the inhibitory signal is conveyed via the striatal complex and its related connections.

It was reported that the micro-infusion of AMPA receptor antagonist into LHb relieved its strong inhibitory effect on the dopamine neurons and evokes a notable elevation in the striatal dopamine release [22]. The present study suggests that over-activity of the dopamine neurons (simulated by tetanic stimulus), especially in the VTA, causes prolonged excitation of LHb PA neurons, which may lead to the suppression in firing rates of these dopamine neurons [3], [20], [21] and hence bring them back to the normal firing rates. Thus, it is considered that the LHb is involved in maintaining the homeostasis of the reward system via a negative feedback mechanism to avoid excessive activities of dopaminergic neurons.

In summary, electrical stimulation of the LHb inhibits the firing of dopamine neurons in VTA and SNpc [3], [21]. However our results from another pathway show that single-pulse and tetanic stimuli of dopaminergic structures evoke a transient suppression and significant elevation in firing of LHb neurons, respectively. Our study suggests that LHb neurons play a potentially critical role, not only in maintaining the efficiency of reward signal encoding through positive feedback process, but also in suppressing over-activity of dopamine neurons via a negative feedback mechanism.

## Materials and Methods

### Animals

Male Wistar rats (220–380 g, mostly 220–250 g) were used for all experiments. They were kept under normal laboratory conditions (temperature 22±2°C, 12 h day-night cycle, lights on at 8 a.m.) with food and water available ad libitum. Rats were anesthetized with 20% urethane (1.2 g/kg, i.p.) and then mounted in a stereotaxic apparatus. Body temperature was maintained at 37°C during the experiments, and mineral oil was applied to the eyes to avoid drying. All procedures were approved by the Local Committee for the Care and Use of Laboratory Animals (SCXK (Ji) 2007-0003 and SYXK (Ji) 2007-0011, 2007) and were conducted in accordance with the guidelines for animal care and use set by local committee.

### Single-unit recording

Glass microelectrodes (impedance of 8–15 MΩ) were filled with 0.5 M NaCl with 2% pontamine sky blue. A hydraulic drive and stepping motor (PC-5N; Narishige, Tokyo, Japan) were used to lower recording electrodes slowly into the LHb (3.3–4.16 mm posterior to bregma, 0.3–1.0 mm lateral to midline, and 4.2–4.6 mm ventral to dura), with the upper incisor bar positioned at interaural zero.

Extracellular potentials were amplified and filtered (0.3–30 kHz bandpass) using a microelectrode amplifier (ME2-8301; Nihon Kohden, Tokyo, Japan), and monitored continuously on a dual-beam storage oscilloscope (VC-10; Nihon Kohden, Tokyo, Japan) and an audio monitor. Discriminated action potentials of LHb neurons were collected and digitized using a data acquisition system (ML-112; ADI, Sydney, Australia); data were stored on disk and analyzed off-line.

### Stimulation parameters

Stainless steel stimulating electrodes were implanted in the anterior VTA (5.3 mm posterior to bregma, 0.8 mm lateral to midline, and 8.2 mm ventral to dura) with the electrode lowered at a 15° angle lateral to the vertical, or in the SNpc (5.3 mm posterior to bregma, 2.4 mm lateral to midline, and 7.2 mm ventral to dura), with the electrode lowered at 5° to the vertical, and were then fixed in place with dental cement. Monophasic and rectangular stimulus pulses (100 µs duration/phase) were generated by an electronic stimulator (SS-102J; Nihon Kohden, Tokyo, Japan) through a stimulus isolation (SEN-7103; Nihon Kohden, Tokyo, Japan) and used for both single-pulse and tetanic stimulation.

For single-pulse stimulation of brainstem dopaminergic nuclei, stimulation parameters used were 0.5 Hz and 300 µA; these values were reported previously to generate phasic dopamine release in the targets of VTA and SNpc [29], [30], [31]. For tetanic stimulation, constant train pulses of 80 µA were applied for 2 s to midbrain nuclei. These parameters had been reported previously to evoke dramatic and long-lasting dopamine release downstream of the VTA and SNpc [38], [39].

### Electrolytic lesions

Lesions of the bilateral fasciculus retroflexus (4.3 mm posterior to bregma, 0.8 mm lateral to midline, and 6.5 mm ventral to dura) were generated using anodal direct current (150 µA for 50 s) via stainless steel electrodes. In general, electrolytic lesion procedures were completed 1 h before the onset of recording. If the proportion of the lesioned fasciculus retroflexus exceeded 90% on average in both sides, it was considered a complete lesion while a lesion that exceeded 60% but not beyond 90% on average was considered a partial lesion.

### Histology

Final tip positions of the recording microelectrodes in the LHb were marked by cathodal current ejection (−15 µA, 30 min) of pontamine sky blue at the end of the recording studies. Electrolytic microlesions were made at midbrain stimulation sites by passing direct current (15 µA for 10 s) through the stimulating electrodes.

After all these procedures were completed, animals were deeply anesthetized with an overdose urethane, and then perfused with saline and 10% neutral-buffered formalin through the left ventricle. The brains were harvested, embedded and stored frozen at −20°C until they were histologically studied. Brain blocks were prepared including the regions of the LHb and midbrain and sectioned on a cryostat (40 µm sections). The locations of pontamine sky blue deposits and electrolytic lesions were determined by microscopic examination of sections after staining with cresyl violet. The Paxinos and Watson atlas of the rat brain [40] was used as a reference.

### Data analysis

When a discriminated single neuron was detected and showed stable firing for a period of 3–5 min, a 2 s tail pinch was applied and the neuron was classified as PA based on a minimal increase in firing rate >20% relative to the return to baseline firing in 1–2 min after tail pinch. Although some cells were studied after only single-pulse or tetanic stimulation, in most cases, cells were studied first with single-pulse and then with tetanic stimulation once baseline firing rates had recovered and stabilized for at least 2–3 min.

Peristimulus time histograms in response to single-pulse stimulations were generated from 100 (or 300) consecutive sweeps with discriminated spike signals accumulated in 1 ms time bins. Phasic increases or decreases in firing rates by >30% from the baseline rate after single-pulse stimulations were defined as activations and suppressions, respectively. Responses to tetanic stimuli were assessed in firing-rate histograms with discriminated spikes accumulated in 5 s time bins; activations and suppressions were defined as >20% increase or decrease, respectively, in firing rate relative to baseline in response to tetanic stimuli.

Independent-sample *t* tests were used to assess comparisons between different groups of latencies and cessation durations in response to single-pulse stimuli, and paired-sample *t* tests were used to assess changes of firing rates in response to tetanic stimuli. Wilcoxon rank-sum or signed-rank tests were adopted, respectively, when the distributions of data did not conform to a normal distribution. Comparison between activated ratios of PA neurons in the LHbM and the LHbL during tetanic VTA stimulus, and comparison between intact and lesioned groups of numbers of responsive neurons to single-pulse stimulation were evaluated with *χ* ^2^ tests. All data were expressed as means ± SEM. and statistical analyses were performed using SPSS 13.0 with the level of statistical significance defined as *P*<0.05 (two-tailed).
