# Correction: Stimulation of Midbrain Dopaminergic Structures Modifies Firing Rates of Rat Lateral Habenula Neurons

**DOI:** 10.1371/annotation/4b2ddb3d-b45e-4a31-9872-4186b9e731a3

**Published:** 2012-05-31

**Authors:** Xuefeng Shen, Xiaoguo Ruan, Hua Zhao

The images for Figures 3 and 4 were incorrectly switched. The image that appears as Figure 3 should be Figure 4, and the image that appears as Figure 4 should be Figure 3. The figure titles and legends appear in the correct order. 

